# Optimisation of Bioluminescent Reporters for Use with Mycobacteria

**DOI:** 10.1371/journal.pone.0010777

**Published:** 2010-05-24

**Authors:** Nuria Andreu, Andrea Zelmer, Taryn Fletcher, Paul T. Elkington, Theresa H. Ward, Jorge Ripoll, Tanya Parish, Gregory J. Bancroft, Ulrich Schaible, Brian D. Robertson, Siouxsie Wiles

**Affiliations:** 1 Department of Medicine, Imperial College London, London, United Kingdom; 2 Department of Infectious and Tropical Diseases, London School of Hygiene and Tropical Medicine, London, United Kingdom; 3 Institute of Electronic Structure and Laser, Foundation for Research and Technology-Hellas, Heraklion, Crete, Greece; 4 Barts and the London School of Medicine and Dentistry, Queen Mary University of London, London, United Kingdom; 5 Infectious Diseases Research Institute, Seattle, Washington, United States of America; 6 Department of Molecular Infection Research, Research Center Borstel, Borstel, Germany; 7 Department of Molecular Medicine and Pathology, University of Auckland, Auckland, New Zealand; Statens Serum Institute, Denmark

## Abstract

**Background:**

*Mycobacterium tuberculosis*, the causative agent of tuberculosis, still represents a major public health threat in many countries. Bioluminescence, the production of light by luciferase-catalyzed reactions, is a versatile reporter technology with multiple applications both in vitro and in vivo. In vivo bioluminescence imaging (BLI) represents one of its most outstanding uses by allowing the non-invasive localization of luciferase-expressing cells within a live animal. Despite the extensive use of luminescent reporters in mycobacteria, the resultant luminescent strains have not been fully applied to BLI.

**Methodology/Principal Findings:**

One of the main obstacles to the use of bioluminescence for *in vivo* imaging is the achievement of reporter protein expression levels high enough to obtain a signal that can be detected externally. Therefore, as a first step in the application of this technology to the study of mycobacterial infection *in vivo*, we have optimised the use of firefly, *Gaussia* and bacterial luciferases in mycobacteria using a combination of vectors, promoters, and codon-optimised genes. We report for the first time the functional expression of the whole bacterial *lux* operon in *Mycobacterium tuberculosis* and *M. smegmatis* thus allowing the development of auto-luminescent mycobacteria. We demonstrate that the *Gaussia* luciferase is secreted from bacterial cells and that this secretion does not require a signal sequence. Finally we prove that the signal produced by recombinant mycobacteria expressing either the firefly or bacterial luciferases can be non-invasively detected in the lungs of infected mice by bioluminescence imaging.

**Conclusions/Significance:**

While much work remains to be done, the finding that both firefly and bacterial luciferases can be detected non-invasively in live mice is an important first step to using these reporters to study the pathogenesis of *M. tuberculosis* and other mycobacterial species *in vivo*. Furthermore, the development of auto-luminescent mycobacteria has enormous ramifications for high throughput mycobacterial drug screening assays which are currently carried out either in a destructive manner using LuxAB or the firefly luciferase.

## Introduction

*Mycobacterium tuberculosis* was first isolated more than 125 years ago. Although a huge amount of research has been devoted to it over this time, tuberculosis still represents a major public health threat in many countries [1]. The main hindrances in fighting this disease include a lack of understanding of the human infection, its establishment and progression, as well as the host-pathogen interactions that determine the different outcomes. Furthermore, the treatment regimen of six months administration of up to four drugs has not evolved in more than four decades, and recent years have seen an alarming increase in multi-drug resistant (MDR) and extensively drug-resistant (XDR) strains. It is clear then that novel and imaginative approaches are needed to speed up both basic and translational research in tuberculosis, especially in the areas of vaccine and drug development.

Bioluminescence, the production of light by luciferase-catalysed reactions, is a versatile reporter technology with multiple applications both *in vitro* and *in vivo*. *In vivo* bioluminescence imaging (BLI) represents one of its most outstanding uses by allowing the non-invasive localisation of luciferase-expressing cells within an animal. Applied to the study of infectious diseases, BLI permits the detection of microorganisms from within living animals thus allowing the spatiotemporal study of infection in real-time in the same host [2]. Moreover, using luciferase as a reporter of gene expression, it is possible to establish when and where a gene function is needed, shedding light on bacterial pathogenesis [3], [4], [5]. Finally, BLI constitutes an easy and rapid method to test novel antimicrobial compounds *in vivo* [6], [7].

Luciferases are a large family of enzymes that catalyse the oxidation of a substrate, generically called luciferin, to yield oxyluciferin with the concomitant production of light. Three main luciferin-luciferase systems have been utilised for BLI [8]. The first system is represented by the firefly luciferase (FFluc) from *Photinus pyralis* which uses D-luciferin (a benzothiazole) as substrate, is dependent on ATP and results in the production of yellow-green light (557 nm). The second system includes the luciferases from the marine organisms *Renilla reniformis* (a cnidarian) and *Gaussia princeps* (a copepod) and the substrate coelenterazine. The signal produced by the *G. princeps* luciferase (Gluc) has been reported to be stronger than that of FFluc [9], even though the light emitted is in the blue range (480 nm) and is therefore more susceptible to tissue absorption and scattering. The fact that Gluc is strongly resistant to heat and extreme pH [9], [10], and that it is secreted by eukaryotic cells also make this system very attractive. Bacterial luciferases, found in the terrestrial bacterium *Photorhabdus luminescens* and marine bacteria from the genera *Vibrio* and *Photobacterium*, constitute the third luciferin-luciferase system. These luciferases are heterodimeric enzymes that use FMNH_2_ and a long-chain aldehyde as substrates. Bacterial luciferases are encoded by the genes *luxAB* that form an operon (*luxCDABE*) together with three additional genes (*luxCDE*) whose products synthesise the long-chain aldehyde. The main advantage of this system is that it does not need exogenously added substrate, but again the light produced is in the blue range (490 nm).

Bioluminescence has been used in mycobacterial research for more than 20 years. Initially, FFluc was used to measure ATP as an indirect method of assessing cell viability and cell numbers [11], [12]. Considering the long duplication times of mycobacteria, the advantages of using bioluminescence as a reporter to assay anti-mycobacterial agents soon became evident when compared to the more traditional colony count methods. To this end LuxAB (requiring the addition of exogenous substrate) and FFluc have been used in *M. smegmatis*, *M. tuberculosis*, *M. bovis* BCG, and even *M. avium*, *M. intracellulare* and *M. aurum* [13], [14], [15], [16], [17], [18], [19]. The development of luciferase reporter phages represented a further improvement as it enabled testing of the drug susceptibility of clinical strains and has subsequently been applied to tuberculosis diagnostics [20], [21], [22], [23], [24], [25], [26], [27]. Finally, luminescent reporter strains have also been used for antibiotic testing and immunity assessment in cell cultures and *ex vivo* in organ homogenates of infected mice [28], [29], [30], [31], [32], [33], [34], [35].

Despite the extensive use of luminescent reporters in mycobacteria, these have only recently been applied to the imaging of mycobacteria *in vivo* [36]. In the single published paper on BLI of mycobacteria, Heus and collaborators utilised a recombinant *M. bovis* BCG strain expressing *luxAB* to monitor mycobacteria infection *in vivo*. While the authors prove that BLI can be used to study bacterial dissemination, drug efficacy and the role of the immune response, their approach has some limitations. In fact, the authors failed to detect BCG in the lungs of infected mice despite being able to detect bacteria in these organs by colony counting and *ex vivo* imaging. Because their luminescent strain of BCG did not express the *luxCDE* genes for substrate synthesis, Heus and collaborators had to administer decanal to the mice in order to image the bacteria. This substrate is highly toxic and, although they were able to deliver it by injection into the murine peritoneum dissolved in a mixture of olive oil and ethanol, this delivery method would appear to have limited distribution to the lungs, which are of major importance for tuberculosis research.

In the work reported here, we have improved the signal obtained from FFluc and Gluc (both of which use non-toxic substrates), and Lux in mycobacteria using a combination of vectors, promoters, and codon-optimised genes. We report for the first time the functional expression of the whole Lux operon in *M. tuberculosis and M. smegmatis* thus allowing the development of auto-luminescent mycobacteria. Moreover, we demonstrate that FFluc and Lux are both useful for the non-invasive detection of mycobacteria in the lungs of infected mice.

## Results

### Maximising luminescent reporter gene expression

**Figure 1:**
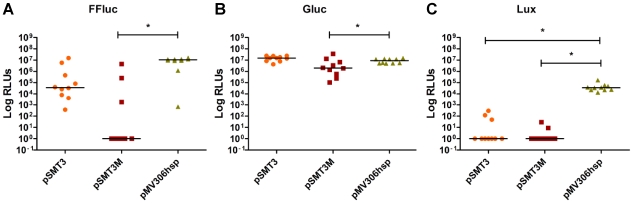
Expression from an integrating vector results in the highest and most stable bioluminescent signal. To study the effect of copy number on light production each reporter was cloned into standard (pSMT3), high (pSMT3M), and single copy number (pMV306hsp) vectors. Luminescence was measured for 10 independent *M. smegmatis* transformants each carrying the FFluc (**a**), Gluc (**b**) and Lux (**c**) constructs. Results are given as relative light units (RLUs) and are corrected for the background. Overall differences between groups were assessed using the Kruskal–Wallis non-parametric test with differences between subgroups assessed by Dunn's multiple comparisons test and those found to be significant (p<0.01) are indicated with *.

As a first step towards the generation of highly-luminescent mycobacteria, each of the three luciferases was cloned into three different expression vectors that contained the same promoter (P*_hsp60_*) but which differ in copy number: (i) pSMT3, which has a pAL5000 replicon and is maintained at 2–5 copies per cell [37], [38]; (ii) pSMT3M [39], which has a mutation in the *repA* gene that increases the copy number to 32–64 copies [40]; and (iii) pMV306hsp which integrates into the chromosomal *attB* site and hence results in single copy number [38]. For the Lux reporter we used a *luxABCDE* operon that had been previously modified for expression in Gram positive bacteria by replacement of the ribosome binding sites and reorganisation of the genes [41]. All the constructs were electroporated into *M. smegmatis*, and the luminescence of 10 randomly selected clones was analysed for each type of construct (Fig. 1). For the three reporters, a high luminescent signal was produced by colonies expressing the integrated constructs derived from pMV306hsp with median values of 1.1×10^7^ RLUs for FFluc, 8.9×10^6^ RLUs for Gluc, and 3.6×10^4^ RLUs for Lux. The pSMT3 derivatives also yielded good signal levels with Gluc (median value, 1.5×10^7^ RLUs) and in three out of ten FFluc clones (4.5×10^5^, 5.8×10^6^ and 1.5×10^7^ RLUs). Remarkably, a high variation was detected among the pSMT3+FFluc clones (signal range of 3.7×10^2^–1.5×10^7^ RLUs), with light production only slightly over the background in three of them (Fig. 1A); this is likely related to the fact that many of these clones carried deletions affecting the reporter gene (data not shown). For all three reporters, the lowest luminescence was obtained with pSMT3M, the high copy number vector. In particular, seven out of ten transformants carrying pSMT3M+FFluc and all the pSMT3M+Lux clones analysed produced only background levels of bioluminescence (Fig. 1A, C). Further screening of 96 pSMT3M+Lux transformants imaged with the IVIS® Spectrum failed to detect any bioluminescent clones. Similarly, out of 387 pSMT3+Lux transformants obtained, only 12 were glowing when imaged with the IVIS® Spectrum. However, these positive clones lost their luminescence after subculturing. Deletions of 5–6 kb, comprising almost the whole *lux* operon, were detected in these and the pSMT3M+Lux transformants (data not shown). Therefore the highest expression and stability was achieved with the pMV306hsp integrating vector, which was selected for further studies.

**Figure 2:**
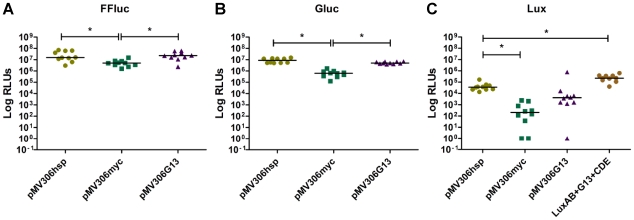
Expression of luciferases using promoters P*_hsp60_* and P_G13_ leads to greater light production. Luminescence of *M. smegmatis* expressing *ffluc* (**a**), *gluc* (**b**) and *lux* (**c**) was assayed using P*_hsp60_*, P*_myc_*tetO, and P_G13_ in the integrating vector pMV306. Each dot represents a randomly selected transformant. Results are given as relative light units (RLUs) and are corrected for the background. Statistical significance was evaluated by the Kruskal–Wallis test with subgroup analysis performed by Dunn's multiple comparison test and those found to be significant (p<0.05) are indicated with *.

Next, we tested different promoters to drive expression of the luciferase genes. Using the integrating vector pMV306, each reporter gene was cloned in front of previously described strong promoters: P*_hsp60_*, P*_myc_*tetO [42], and P_G13_ [43], [44]. These constructs were introduced into *M. smegmatis*, and the luminescence of 10 randomly selected transformants analysed (Fig. 2). Similar luminescence values were obtained among strains expressing either FFluc or Gluc under the control of either P*_hsp60_* or P_G13_ (median values of 1.5×10^7^ and 2.3×10^7^ RLUs respectively for FFluc, 8.9×10^6^ and 5.05×10^6^ RLUs for Gluc), while production of light from P*_myc_*tetO clones was 3–13 times lower. In the case of Lux, the highest luminescence was achieved using P*_hsp60_* (3.6×10^4^ RLUs), followed by P_G13_ (9 times lower), and P*_myc_*tetO (180 times lower than that of P*_hsp60_*). Consequently P*_hsp60_* was the promoter chosen for expression of the luminescent reporters. Additionally, in order to increase the amount of substrate synthesised by the Lux operon and in this way the amount of luminescence, P_G13_ was cloned in front of *luxC* in pMV306hsp+Lux. This resulted in a 6-fold increase in the luminescence activity compared to the original pMV306hsp+Lux, from 3.6×10^4^ to 2.25×10^5^ RLUs (Fig. 2C).

**Figure 3:**
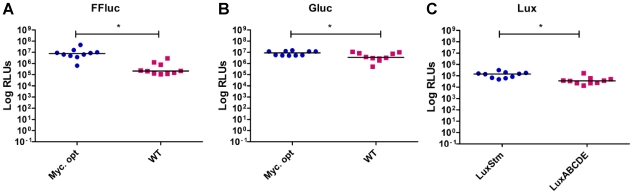
Codon optimisation increases bioluminescence *in vitro*. Relative light units (RLUs) were measured in 10 *M. smegmatis* clones transformed with a wild-type or a *Mycobacterium* optimised FFluc (**a**) or Gluc (**b**), or with a Gram positive or *Streptomyces* optimised Lux (**c**). In all cases pMV306hsp was used as backbone. Results are corrected for the background. Statistical significance was evaluated by the Mann-Whitney non-parametric test for FFluc and Lux, and by unpaired t test for Gluc (data normality passed) and those found to be significant (p<0.05) are indicated with *.

Lastly, as a potential way of enhancing the expression of reporter genes at the translational level, we utilised versions of *ffluc* and *gluc* whose codon usage had been optimised for use in mycobacteria, together with an improved Shine Dalgarno sequence [45]. We also tested a *lux* operon with codon usage optimised for mycobacteria, but no luminescence was produced by this strain, even if the substrate for the bacterial luciferase was added exogenously (data not shown). Alternatively, we used a *luxCDABE* operon (LuxStm) codon-optimised for *Streptomyces coelicolor* [46]. Using pMV306hsp as the expression vector the luminescence of the optimised reporters was compared to that of the wild-type genes (Fig. 3). Codon optimisation resulted in a 30-fold, 2.5-fold and 4-fold improvement in the signal for FFluc, Gluc and Lux respectively. Accordingly, the optimised *gluc* and *ffluc* genes were selected for further study. However, since the luminescence of LuxStm was lower than that of the reorganised *lux* operon with P_G13_ cloned in front of *luxC,* the latter was chosen for further study.

**Figure 4:**
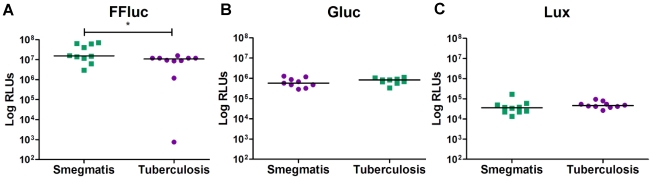
Bioluminescence levels in *M. tuberculosis* and *M. smegmatis* are comparable *in vitro*. Relative light units (RLUs) were measured in 10 *M. smegmatis* and 10 *M. tuberculosis* clones transformed with pMV306hsp+FFluc (**a**), pMV306hsp+Gluc (**b**) or pMV306hsp+Lux (**c**). Results are corrected for the background. Statistical significance was evaluated by the Mann-Whitney non-parametric test for Lux, and by unpaired t test for FFluc and Gluc (data normality passed) and those found to be significant (p<0.05) are indicated with *.

The three reporters were also tested in *M. tuberculosis* by electroporating pMV306hsp+FFluc, pMV306hsp+Gluc and pMV306hsp+Lux and measuring the bioluminescence of 10 randomly selected clones (Fig. 4). The signal obtained in *M. tuberculosis* was slightly lower than that of *M. smegmatis* for FFluc (1.1×10^7^ and 1.5×10^7^ RLUs, respectively), whereas no significant differences were detected for Gluc and Lux (5.8×10^5^ vs. 8.4×10^5^ RLUs, and 3.6×10^4^ vs. 4.6×10^4^ RLUs, correspondingly).

### Optimisation of the *in vitro* bioluminescence reaction

**Figure 5:**
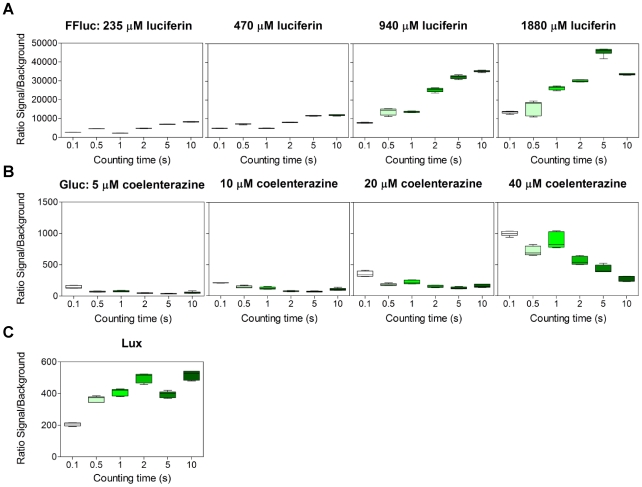
Signal:noise for FFluc and Lux increases with integration time, and decreases for Gluc. Luminescence was measured using six different integration times and four different substrate concentrations for *M. smegmatis* producing FFluc (**a**) and Gluc (**b**), or without substrate for Lux (**c**). The background luminescence was obtained from cultures of *M. smegmatis* with the empty pMV306hsp. Assays were performed with three independent mid-log cultures and each culture was measured in duplicate. As the data was not normally distributed, median values are displayed (bar) with inter-quartile ranges (box), and highest and lowest values (whiskers).

To achieve the highest signal to background ratio we tested different integration times (0.1–10 s) for the three reporters, at four different substrate concentrations for Gluc and FFluc (Fig. 5). As shown in Fig. 5A, increasing the integration time yielded an increased ratio for FFluc, reaching a maximal ratio at integration times between 5 and 10 s, depending on substrate concentration. This was also the case for Lux, with 10 s yielding the best result (Fig. 5C). In contrast, increasing the integration time resulted in a decreased signal to noise ratio for Gluc (Fig. 5B). This was likely due to the high background produced by auto-oxidation of its substrate coelenterazine [47]. Therefore, for further studies the integration times selected were 5 s, 0.1 s and 10 s for FFluc, Gluc and Lux respectively.

**Figure 6:**
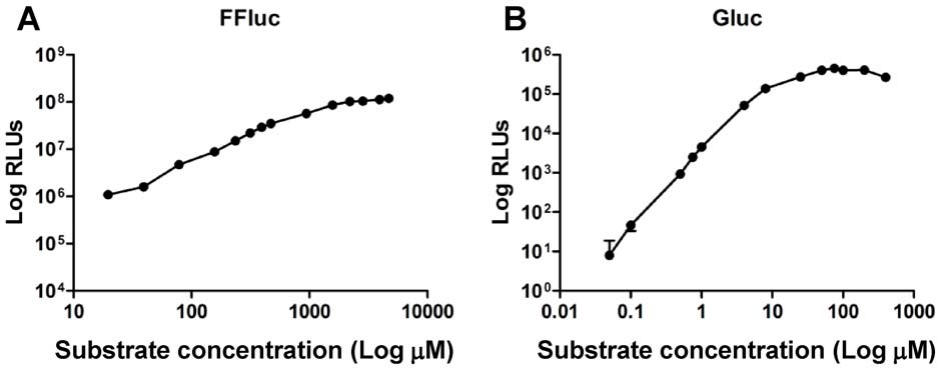
Bioluminescence correlates with substrate concentration at low concentrations. Luminescence (given as relative light units [RLUs]) of *M. smegmatis* pMVhsp+FFluc (**a**) and *M. smegmatis* pMVhsp+Gluc (**b**) was measured with integration times of 5 s and 0.1 s respectively. The substrate concentrations assayed ranged from 20 to 4710 µM luciferin for FFluc, and from 0.05 to 400 µM coelenterazine for Gluc. Means and standard deviations (smaller than symbols) of six replicates are shown.

We then studied the effect of substrate concentration on light production by FFluc and Gluc (Fig. 6). In both cases the luminescence increased with increasing substrate concentration in an exponential manner until a plateau was reached at 40 µM coelenterazine for Gluc and 1570 µM luciferin for FFluc, with 40 µM and 470 µm chosen as working concentrations.

### Luminescence reaction kinetics

**Figure 7:**
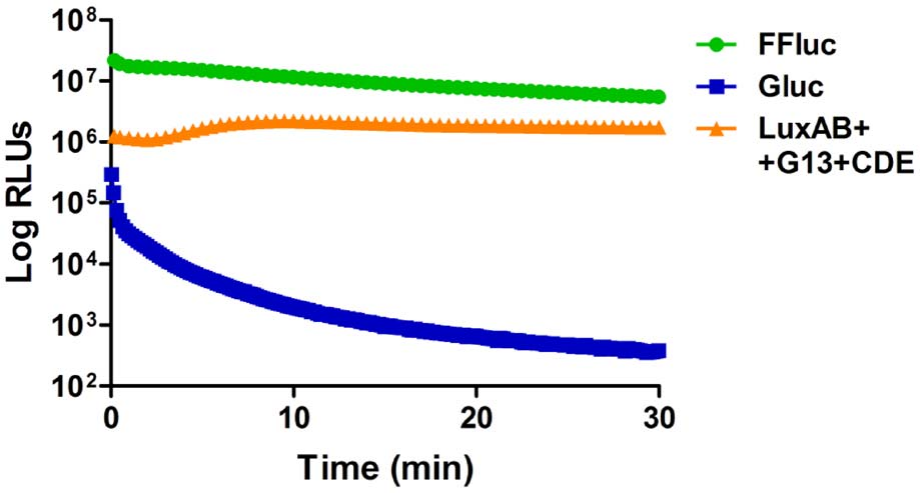
Light production from FFluc and Lux is stable, whereas the signal from Gluc rapidly dissipates. Luminescence (given as relative light units [RLUs]) was measured every 10 s for *gluc*-expressing *M. smegmatis* and every 30 s for *ffluc*- or *lux*-expressing *M. smegmatis*, over a 30 min period. The integration times used were 0.1 s, 5 s and 10 s for Gluc, FFluc and Lux respectively. At time point 0 min, 470 µM luciferin or 40 µM coelenterazine were added to FFluc- and Gluc-producing *M. smegmatis* respectively. Two independent cultures were used for FFluc and Lux, and three for Gluc. Each culture was measured in duplicate and the means and standard deviations (smaller than symbols) are plotted.

The kinetics of light output for each luciferase was measured for *M. smegmatis* pMVhsp+FFluc, pMVhsp+Gluc and pMVhsp+LuxAB+G13+CDE over 30 min, following the addition of substrate as appropriate (Fig. 7). For both Gluc and FFluc, the maximum signal was obtained immediately after substrate addition with median values of 2.9×10^5^±8.9×10^3^ RLUs for Gluc and 2.2×10^7^±7.1×10^5^ RLUs for FFluc. Then, Gluc luminescence decreased dramatically with a 90% loss in just 1 min, whereas the FFluc signal dropped slowly, still retaining 50% of the initial signal after 17 min. In contrast, the light output for Lux remained stable between 1.1×10^6^ and 2.2×10^6^ RLUs during the whole experiment, most likely reflecting a continuous production of substrate by the cells.

**Figure 8:**
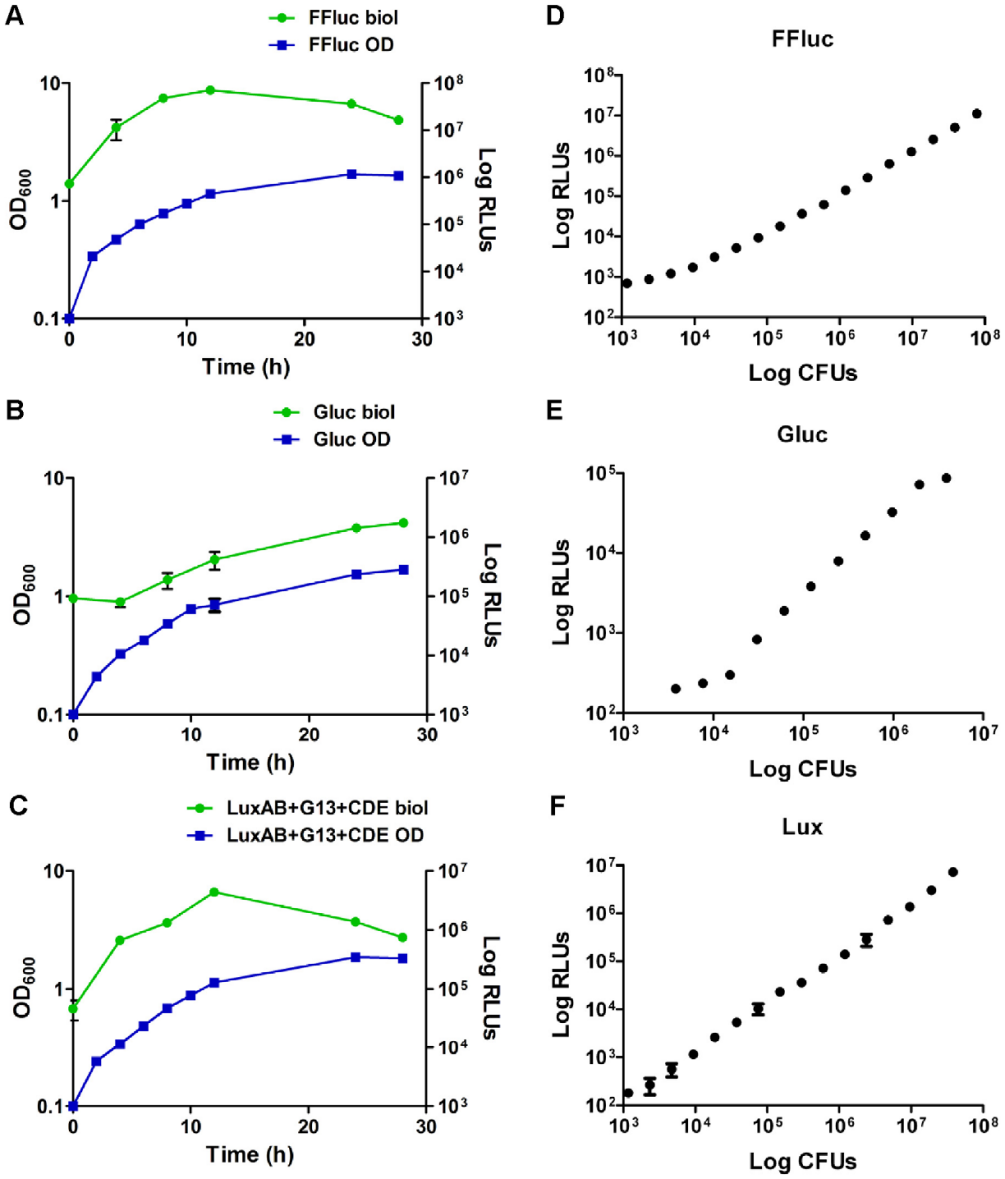
Bioluminescence correlates with cell density during exponential growth *in vitro*. Cultures of *M. smegmatis* pMVhsp+FFluc (**a**), pMVhsp+Gluc (**b**) and pMVhsp+Lux (**c**) were inoculated to an optical density (OD) at 600 nm of 0.1 and the OD and the luminescence [given as relative light units (RLUs)] measured over 28 h. The luminescence was measured with integration times of 5, 0.1, and 10 s respectively, and substrate concentrations of 470 µM luciferin for FFluc and 40 µM coelenterazine for Gluc. The values represented correspond to the means of two independent cultures measured in triplicate. The error bars indicate standard deviations. A near linear relationship was found between bioluminescence [given as RLUs] and colony counts (given as colony forming units [CFU]) for mid-log cultures of *M. smegmatis* pMVhsp+FFluc (**d**), pMVhsp+Gluc (**e**) and pMVhsp+Lux (**f**) using a plate luminometer.

We then analysed luminescence production during the growth of the same luciferase-producing strains (Fig. 8). The level of luminescence correlated well with cell density during the exponential growth phase for all the three reporters. However, the signal dropped when the cultures of *lux*- and *ffluc*-expressing cells entered stationary phase with a total loss of 83% and 77% respectively between the time points 12 and 28 h (Fig. 8A, C). This is most likely related to a decrease in the metabolic activity of the bacteria, and therefore in the availability of the FMNH_2_ and ATP needed by Lux and FFluc, respectively. In contrast, luminescence from cells expressing the *Gaussia* luciferase, which does not require any bacterial cofactors to catalyse the luminescence reaction, remained related to cell numbers until the end of the growth curve (Fig. 8B). Moreover, using mid-log cultures a good correlation (Spearman r = 1) was found between colony forming units (CFU) and luminescence (measured with the plate luminometer) over a range of cell numbers: 10^4^ and 10^8^ CFU for *ffluc*-expressing *M. smegmatis*, 10^3^ and 10^8^ for *lux*-expressing cells, and 10^4^–10^6^ for Gluc producing *M. smegmatis* (Fig. 8D–F).

### Secretion of *Gaussia* luciferase

**Figure 9:**
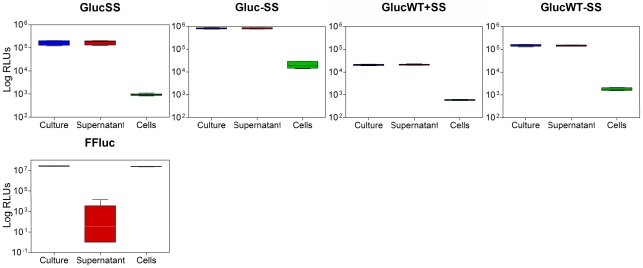
*Gaussia* luciferase is secreted from mycobacterial cells. Luminescence (given as relative light units [RLUs]) was measured in culture, supernatant and cell samples of *M. smegmatis* producing Gluc *Mycobacterium* optimised with (GlucSS) or without (Gluc) signal peptide, Gluc wild-type with (GlucWT+SS) or without (GlucWT-SS) signal peptide, and FFluc as control. Assays were performed with three independent cultures and each culture was measured in duplicate. As the data was not normally distributed, median values are displayed (bar) with inter-quartile ranges (box), and highest and lowest values (whiskers).

It has previously been stated that Gluc is secreted from eukaryotic cells because of a signal peptide situated at the N-terminal end of the protein [9]. To determine if Gluc was also secreted in *M. smegmatis*, we examined the luminescence produced by the whole culture, the cells or the culture supernatant. Moreover, as a way to assess the role of the signal peptide, experiments were also performed using strains expressing codon-optimised and wild-type forms of Gluc without the signal sequence. As shown in Fig. 9, almost 100% of the luminescence was detected in the supernatant of all *gluc*-expressing *M. smegmatis* regardless of the presence or absence of the signal peptide and the codon usage of the gene used. In contrast, only 2% of the total luminescence was found in the supernatant of FFluc-producing *M. smegmatis* when analysed in a similar way. Surprisingly, the light output from Gluc was higher when the signal peptide was deleted (Fig. 9), both for the *Mycobacterium* optimised (8.2×10^5^±5.6×10^4^ RLUs compared to 1.75×10^5^±3.8×10^4^ RLUs with signal peptide) and the wild-type genes (1.5×10^5^±1.36×10^4^ RLUs compared to 2.1×10^4^±1.3×10^3^ RLUs).

### Bioluminescence *in vivo* imaging

To assess if the signal produced by each of the three reporters could be detected *in vivo*, three or four Balb/c mice were endotracheally inoculated with 10^6^–10^7^ CFU of luminescent *M. smegmatis* and imaged 24 h later with the IVIS® Spectrum. The background level of luminescence was estimated by imaging two mice inoculated with the non-luminescent *M. smegmatis* pMV306hsp.

**Figure 10:**
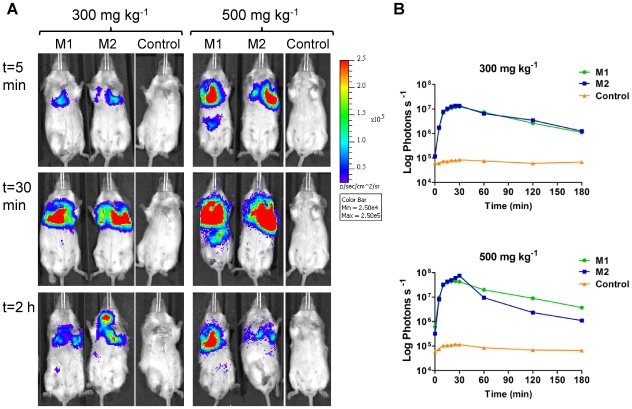
Kinetics of FFluc activity in *M. smegmatis* infected mice after intraperitoneal injection of luciferin. Mice were endotracheally inoculated with 1.4×10^7^ CFU of *M. smegmatis* pMV306hsp+FFlucWT [two representative mice (M1 and M2) out of four are shown] or with 6.8×10^6^ CFU of *M. smegmatis* pMV306hsp (control). 300 mg kg^−1^ or 500 mg kg^−1^ luciferin intraperitoneal was given 24 h post-inoculation and images were acquired at time points 0 (immediately after substrate administration), 5, 10, 15, 20, 25, 30, 60, 120 and 180 min. (**a**) Images were obtained using an IVIS Spectrum and are displayed as pseudocolour images of peak bioluminescence (given as photons s^−1^ cm^−2^ steridian [sr]^−1^), with variations in colour representing light intensity at a given location. Mice injected with 300 mg kg^−1^ luciferin were imaged with an integration time of 1 min, whereas those that received 500 mg kg^−1^ luciferin were imaged for 10 s to avoid saturation of the image. Three representative time points are shown. (**b**) Bioluminescence (given as photons s^−1^) in the thorax was quantified for each time point using the region of interest tool in the Living Image software program.

Two substrate concentrations and two routes of administration were assessed for FFluc: (i) 300 mg kg^−1^ body weight and 500 mg kg^−1^ body weight for the intraperitoneal route, and (ii) 15 mg ml^−1^ and 30 mg ml^−1^ for the intranasal route. Images were acquired over a 3 h time period to determine the optimal time to image after luciferin administration. For luciferin administered via the intraperitoneal route, no signal could be detected until 5 min post-substrate administration at which point bioluminescence was evident in the lungs of all four positive mice and in the abdomen of one mouse injected with 500 mg kg^−1^ luciferin (Fig. 10). The signal in the abdomen most likely reflects the presence of *ffluc*-expressing *M. smegmatis* in the gastrointestinal tract, which could be due to the introduction of some bacteria into the oesophagus during the endotracheal inoculation. After the first 5 min, the bioluminescent signal increased exponentially, reaching a peak 25–30 min after substrate injection, with a level four to six times greater when using the higher concentration of luciferin (4.5×10^7^–7.5×10^7^ photons s^−1^ compared to 1.2×10^7^–1.4×10^7^ photons s^−1^). After that the light level diminished approximately 50% in 30 min, followed by another 50% decrease every hour until the end of the experiment. No signal was detected in mice inoculated with *M. smegmatis* pMV306hsp (Fig. 10A), with stable background levels during the whole experiment (6×10^4^–8×10^4^ photons s^−1^ for 300 mg kg^−1^ luciferin, and 5.9×10^4^–1.2×10^5^ photons s^−1^ for 500 mg kg^−1^ luciferin, Fig. 10B). Therefore the best conditions for imaging of *ffluc*-expressing *M. smegmatis* given intraperitoneal substrate are using 500 mg kg^−1^ luciferin and imaging 25–30 min post-substrate administration.

**Figure 11:**
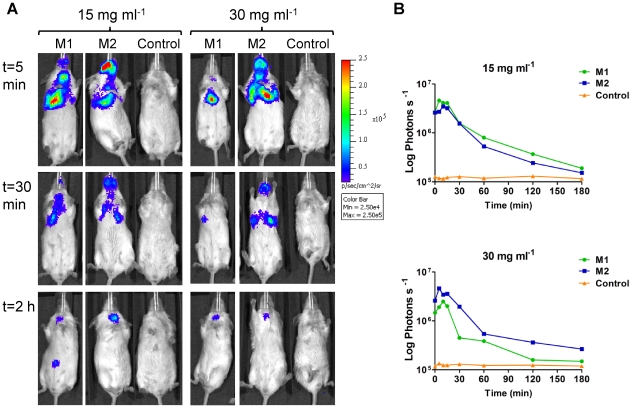
Kinetics of FFluc activity in *M. smegmatis* infected mice after intranasal administration of luciferin. Mice were endotracheally inoculated with 6.6×10^6^ CFU of *M. smegmatis* pMV306hsp+FFlucWT [two representative mice (M1 and M2) out of four are shown] or with 6.8×10^6^ CFU of *M. smegmatis* pMV306hsp as a control (one representative mouse out of two is shown). 20 µl of 15 mg ml^−1^ or 30mg ml^−1^ luciferin intranasal was administered 24 h post-inoculation and mice were imaged 0, 5, 10, 15, 30, 60, 120 and 180 min after. (**a**) Images were obtained using an IVIS Spectrum and are displayed as pseudocolour images of peak bioluminescence (given as photons s^−1^ cm^−2^ sr^−1^). Red represents the most intense light emission while blue correspond to the weakest signal. The colour bar indicates relative signal intensity. Mice were imaged with an integration time of 30 s. Three representative time points are shown. (**b**) Signal intensity (given as photons s^−1^) in the lungs was quantified for each time point using the region of interest tool in the Living Image software program.

For luciferin administered by the intranasal route, bioluminescence could be detected immediately after substrate introduction (Fig. 11), and the maximum signal in the lungs was obtained in just 5–10 min (3.5×10^6^–4.6×10^6^ photons s^−1^ when 15 mg ml^−1^ luciferin was administered, and 2.5×10^6^–4.6×10^6^ photons s^−1^ for 30 mg ml^−1^ luciferin). In addition to the signal in the lungs a strong signal could be detected in the nose of the three positive mice (Fig. 11A) indicating the presence of FFluc-producing *M. smegmatis* in that organ too. This signal was not observed when the luciferin was administered via the intraperitoneal route, most likely as a result of the increased systemic distribution of the substrate. After reaching a peak, the bioluminescence in the lung and nose decreased by 50–80% in 30 min falling close to background levels 2–3 h after substrate administration (Fig. 11). The background levels remained constant during the whole experiment at approximately 1.2×10^5^ photons s^−1^. Thus the best conditions for imaging of *ffluc*-expressing *M. smegmatis* given intranasal substrate are using 15 mg ml^−1^ luciferin and imaging 5–10 min post-substrate administration.

**Figure 12:**
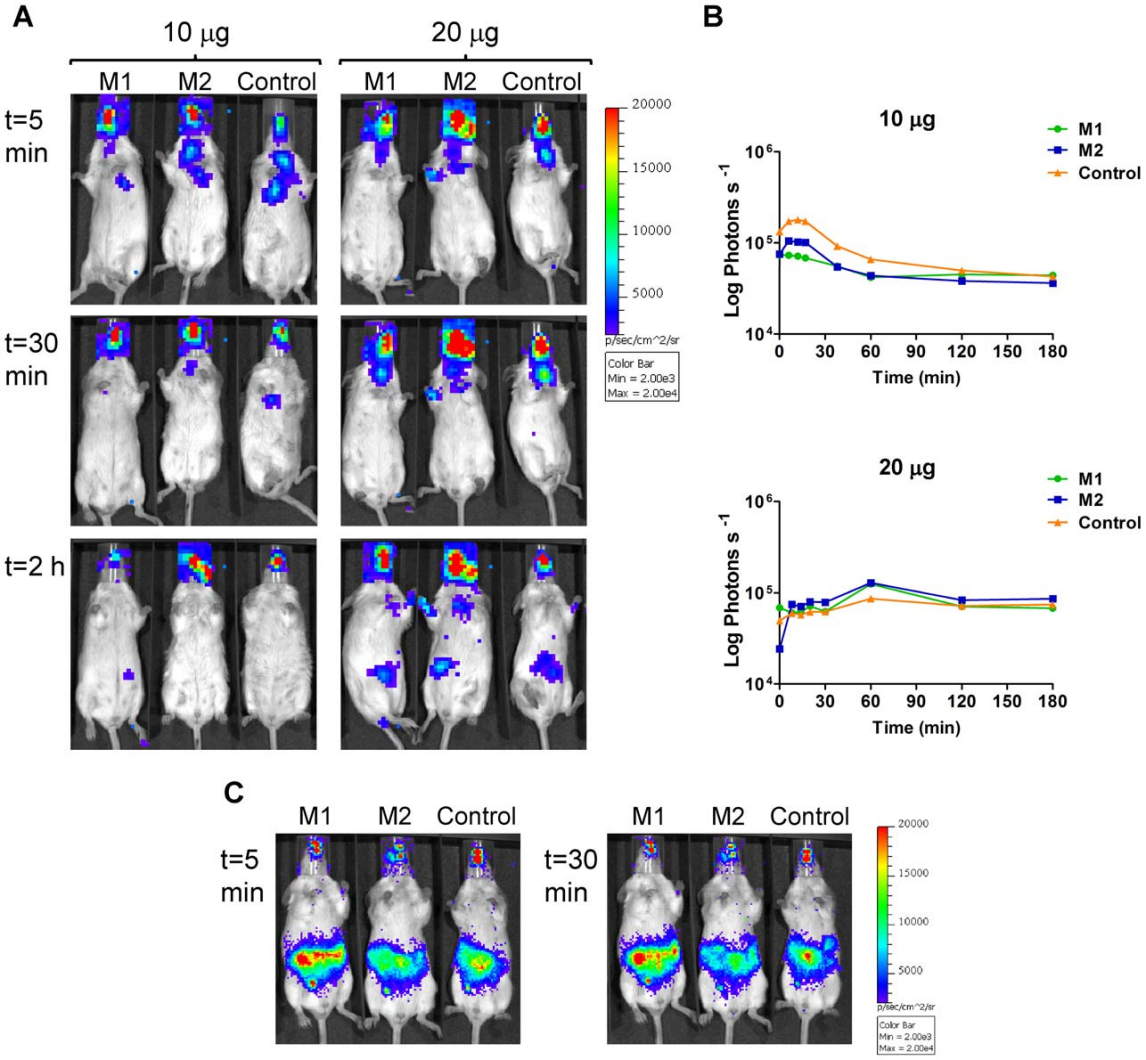
BLI of *gluc*-expressing *M. smegmatis*. Mice were endotracheally inoculated with 3.32×10^6^ CFU of *M. smegmatis* pMV306hsp+Gluc [two representative mice (M1 and M2) out of three are shown,) or with 1.58×10^7^ CFU of *M. smegmatis* pMV306hsp as a control (one out of two mice is shown). 10 µg of coelenterazine intranasal was administered 24 h post-inoculation and mice were imaged at time points 0, 5, 10, 15, 30, 60, 120 and 180 min. (**a**) Images were obtained using an IVIS Spectrum and are displayed as pseudocolour images of peak bioluminescence (given as photons s^−1^ cm^−2^ sr^−1^), with variations in colour representing light intensity at a given location. Integration time was 5 min. (**b**) Bioluminescence (given as photons s^−1^) was quantified using the Living image software. (C) 10 µg of coelenterazine was given intraperitoneally to the same mice 5 h post-intranasal coelenterazine. Mice were imaged 0, 5, 10, 15, 20, 25 and 30 min post-intraperitoneal coelenterazine with integration times of 3 min.

A similar assay was performed using Gluc. Two coelenterazine concentrations were administered intranasally (10 and 20 µg) and images were acquired at different time points over 3 h. A high background was detected in all cases and no differences were observed between mice inoculated with *M. smegmatis* pMV306hsp+Gluc or *M. smegmatis* pMV306hsp (Fig. 12A, B). The same mice were then administered 10 µg coelenterazine by the intraperitoneal route 5 h after initial intranasal substrate administration. A high background signal was detected in the abdomen of both the positive and control mice (Fig. 12C), while the bioluminescence observed in the nose was due to the previous intranasal coelenterazine. Consequently, Gluc is not useful for *in vivo* imaging of *M. smegmatis*.

**Figure 13:**
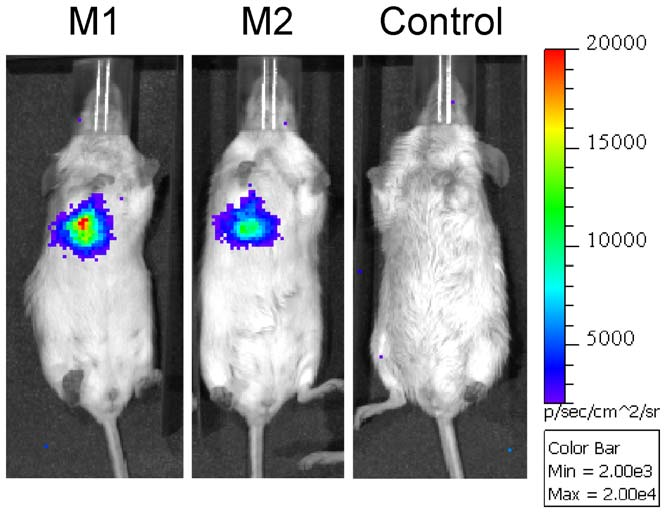
BLI of *lux*-expressing *M. smegmatis*. Mice were inoculated endotracheally with *M. smegmatis* pMV306hsp+LuxAB+G13+CDE [7.9×10^6^ CFU, two mice (M1 and M2) out of four are shown] or *M. smegmatis* pMV306hsp (6.8×10^6^ CFU, control) and imaged 24 h post-inoculation. Images were obtained using an IVIS Spectrum and are displayed as pseudocolour images of peak bioluminescence (given as photons s^−1^ cm^−2^ sr^−1^), with variations in colour representing light intensity at a given location. Mice were imaged with an integration time of 5 min.

Mice infected with *M. smegmatis* pMVhsp+LuxAB+G13+CDE were also imaged. Bioluminescence could be detected in the lungs of the positive mice while no signal was observed in the control mouse (Fig. 13).

**Figure 14:**
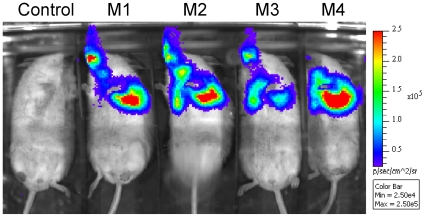
BLI of *ffluc*-expressing *M. tuberculosis*. Mice were inoculated endotracheally with 5×10^6^ CFU of either wild-type *M. tuberculosis* (control) or FFluc-producing *M. tuberculosis*. 20 µl of 30 mg ml^−1^ luciferin was administered intranasally and mice were imaged 5–10 min after. Mice were contained in a large air-tight box for safety considerations. The image was obtained using an IVIS Spectrum and is displayed as a pseudocolour image of peak bioluminescence (given as photons s^−1^ cm^−2^ sr^−1^). Red represents the most intense light emission while blue correspond to the weakest signal. The colour bar indicates relative signal intensity. Mice were imaged with an integration time of 1 min.

Finally, we also imaged mice infected with *ffluc*-expressing *M. tuberculosis* after administering intranasal luciferin. Bioluminescence was detected in the lungs of mice receiving *ffluc*-expressing bacteria, while no signal was observed in the control mouse infected with the wild-type *M. tuberculosis* H37Rv (Fig. 14).

## Discussion

Work with mycobacteria is hampered by their long duplication times meaning that, in the case of *M. tuberculosis,* results based on organ CFU counts are only available three to four weeks after the conclusion of the experiment. This handicap can, in part, be overcome by the use of non-invasive imaging techniques which allow infection dynamics to be studied in real-time. Moreover, such techniques allow for drastic reductions in the numbers of animals used in experiments. Recently an *M. tuberculosis* recombinant strain expressing a bacterial thymidine kinase was visualised *in vivo* using [^125^I]FIAU-single photon emission computed tomography (SPECT) [48]. While this technique is able to accurately measure the level of signal and has good spatial resolution, there are some issues with non-specific signals and the relatively high cost [48].

An alternative to this technique is the use of BLI which has proved very useful in the study of infectious diseases caused by a variety of bacteria [49], [50], [51], [52], [53], [54]. In the only work on BLI of mycobacteria published so far, the *luxAB* genes were used, thus requiring the administration of the toxic substrate decanal. Although the infection could be detected in the abdomen of intravenously infected mice, the signal in the lungs was too low to be detected after intraperitoneal administration of aldehyde. To overcome these problems and thus further develop BLI imaging of mycobacteria, we have optimised the FFluc and Gluc luciferases for use in mycobacteria, both of which use non-toxic substrates, and the whole *lux* operon that does not require the external addition of substrate.

Approaches for imaging reporter gene expression mainly depend on robust levels of reporter protein. This, in turn, depends on the nature of the expression vector used, including the copy number, and the transcriptional and translational signals. With this in mind we compared the bioluminescent signal obtained with each reporter using three different vectors (two episomal vectors with 2–5 and 32–64 copies per cell, and one integrating vector), three different promoters (P*_hsp60_*, P*_myc_*tetO, and P_G13_), and finally, with an optimised Shine-Dalgarno sequence, we also tested the effect of codon optimisation. Our results demonstrate that the best expression was achieved using the integrating vector, a finding most likely linked to a considerable instability of the replicating vectors when expressing *ffluc* and *lux*. In fact, the whole *lux* operon was only stably expressed in *M. smegmatis* when it was cloned into the integrating vector, with no positive clones isolated using the episomal vectors. This would explain why this reporter operon had never been successfully expressed in mycobacteria until now. A similar situation has also been reported for the expression in mycobacteria of other recombinant genes like the HIV-1 gp120 [38] or the human interleukin 2 [55]. The more stable expression of foreign antigens in mycobacteria using integrating vectors as opposed to episomal vectors has been extensively documented [56], [57], [58], [59] and is mainly attributed to lower expression, and therefore a lower metabolic burden, associated with the reduction in the copy number.

In the case of the *lux* operon, we have previously found that plasmid-based expression of *luxD* is toxic in mycobacteria (S. Wiles, unpublished results). This gene encodes an acyl transferase which forms part of an enzyme complex (along with the products of *luxC* and *luxE*) responsible for recycling the fatty acid by-products of the luciferase reaction back into long chain aldehydes for use as further substrate. Given the importance of fatty acids in the cell wall structure of mycobacteria, it is not surprising that over-expression of fatty acid modifying enzymes would be detrimental.

Regarding promoter selection, we have found that the promoter P*_hsp60_* drove the highest luminescence for the three reporters, although the differences between the three promoters were only minor. P*_hsp60_* is known to be a strong promoter in mycobacteria and has been used extensively to over-express proteins [38], [60], [61], [62], [63]. However, P*_myc_*tetO, isolated from an *M. smegmatis*/*M. tuberculosis* library using GFP to assess gene expression *in vitro* [42]; and P_G13_, isolated from an *M. marinum* library expressing GFP inside macrophages [43], [44], have both been reported to be stronger than P*_hsp60_* (10 and 10–20 times, respectively). The discrepancy with our results could be related to the different reporters used to measure gene expression, and the toxicity and metabolic load associated with their over-expression. It may also be that the highest stable level of expression for our reporters had already been achieved with the P*_hsp_* and therefore the use of stronger promoters did not increase expression further. This would be in agreement with the instability observed using the episomal vectors for *ffluc* and *lux*, and when using the P*_myc_*tetO with *lux*.

Perhaps unsurprisingly, codon-optimisation was found to increase the signal obtained for FFluc (30-fold [76% homology]) and Gluc (2.5-fold [74% homology]). Unexpectedly, the *M. tuberculosis* codon-optimised Lux was found to be non-functional, even after the addition of exogenous substrate. We have not explored the basis for this inactivity; however, there is a suggestion from the optimised sequence that after transcription the DNA may form secondary structures that impede translation (data not shown).

We also characterised the various bioluminescence reactions by varying the integration time and substrate concentration, and by measuring the kinetics of light output. The results obtained are similar to those previously described [9], [10] proving that Gluc catalyses a rapid ‘flash’ reaction, FFluc has glow kinetics, and the continuous synthesis of substrate by the *lux* operon allows for steady luminescence. In this work, we have found that FFluc produced the highest luminescence, 10 times brighter than that obtained with Lux, and 100 times that of Gluc. This is in contrast with the results of Snewin et al. who found LuxAB to be brighter than FFluc [31], but this was done using an episomal reporter that required the addition of aldehyde substrate. Consequently, the substrate may have been in excess, which might be a limiting factor in our whole operon *lux* reporter. To test this hypothesis an extra promoter was cloned in front of *luxCDE* to boost substrate synthesis, and indeed a 6-fold higher signal was obtained. The alternative of adding exogenous substrate also increased the signal 5–10 times (data not shown). The availability of FMNH_2_ could also be a limiting factor. In yeast, co-expression of *luxAB* together with the *frp* gene, encoding a NADPH-FMN oxidoreductase from *Vibrio harveyi*, led to a 100-fold increase in the luminescence [64]. However, we have found the same approach in mycobacteria to have no effect on the final signal (unpublished results).

Once the best conditions for reporter expression were selected, we proceeded to explore if the bioluminescence signal obtained was strong enough for the imaging of mycobacteria *in vivo*. Importantly, bioluminescence could be detected in the lungs of mice infected with either *ffluc* or *lux*-expressing *M. smegmatis* or *ffluc*-expressing *M. tuberculosis*. FFluc utilises a non-toxic, well tolerated substrate that can be repeatedly administered for repetitive imaging. The main route of luciferin administration is by intraperitoneal injection, however intranasal delivery has been recently described as a more efficient route for the monitoring of bioluminescence in the lungs, while using 30 times less luciferin [65]. We have found that a good signal is obtained using intranasal luciferin, although a stronger signal can be achieved using intraperitoneal luciferin which would also have the advantage of allowing detection of signal in other locations besides the lungs.

Surprisingly, the signal from Gluc-producing *M. smegmatis* in mice could not be distinguished from the strong background produced by the coelenterazine substrate alone. These results were unexpected considering that published work with eukaryotic cells states that the Gluc signal is 1000 times stronger than that of FFluc in cell culture, and as bright as FFluc *in vivo*, with no background detected *in vivo* even using a 20-fold higher concentration of coelenterazine [9]. Gluc is naturally secreted from eukaryotic cells and this has been used to quantify the number of Gluc-producing cells in murine blood samples [66]. Although we have proved that Gluc is also secreted from *M. smegmatis*, we have been unable to detect it in blood samples taken from infected mice thus far (data not shown). Overall these results preclude Gluc as a reporter for either *in vivo* imaging of mycobacteria or *ex vivo* monitoring of infected organs.

The results presented here represent an important step towards the use of bioluminescence for the non-invasive study of mycobacteria infection *in vivo*. In fact, the use of FFluc, or the whole *lux* operon would represent a major advantage compared, for example, to the use of just *luxAB*. There are, however, some issues that should be considered and further investigated. First, the minimal number of bacteria capable of being detected within the lungs is expected to be quite high, especially when compared to the inocula that are normally used in experimental *M. tuberculosis* infections. Although this could limit the use of BLI studies for the initial stages of the infection, it would not pose a problem in more advanced phases of the infection and would still represent an important improvement in drug or vaccine efficacy testing. Secondly, luciferin accessibility to the bacteria contained in granulomatous lesions could affect the signal obtained and has to be further investigated. To that end, preliminary results have shown that luminescence can be detected during infection of macrophage *in vitro* thus proving the permeability of macrophages to luciferin (unpublished data). Finally, the signal obtained is also affected by the metabolic state of the bacteria since the luciferase reaction relies on bacterial metabolites, mainly ATP and FMNH_2_ for FFluc and Lux respectively. This is exemplified by the decrease in the luminescence observed when *M. smegmatis* enters the stationary phase and has also been previously described for other bacteria [67], [68], [69], [70], [71]. Although this could represent a handicap for the study of, for example, dormant *M. tuberculosis* it could be a powerful tool to gain insights into the metabolic state of *M. tuberculosis* during infection, to study entrance into dormancy, as well as for the rapid detection of drugs acting on bacterial metabolism.

## Materials and Methods

### Bacterial strains and growth conditions

*M. smegmatis* mc^2^155 [72], *M. tuberculosis* H37Rv and *Escherichia coli* NEB-10β (New England Biolabs UK Ltd) were used in this work. *M. smegmatis* and *M. tuberculosis* were grown on Middlebrook 7H11 agar medium (BD Diagnostics) supplemented with 0.5% glycerol and 10% oleic acid albumin-dextrose-catalase (OADC) (BD Diagnostics). When required, filter-sterilised luciferin was added at a final concentration of 0.157 mM. Liquid cultures of *M. smegmatis* and *M. tuberculosis* were grown either in Middlebrook 7H9 broth (BD Diagnostics) containing 0.05% Tween 80 (Sigma) and 10% albumin-dextrose-catalase (ADC) enrichment (BD Diagnostics), or (for *M. smegmatis* Gluc assays) in Luria-Bertani (LB) medium with 0.05% Tween. LB medium was preferred for the Gluc assays because the background of coelenterazine was 100 times lower in that medium than in 7H9 broth. LB medium was used for culturing *E. coli*. All the strains were grown at 37°C. The following antibiotics were added when appropriate: ampicillin [100 µg ml^−1^ (Sigma)], hygromycin B [150 µg ml^−1^ (Invitrogen)] and kanamycin [25 µg ml^−1^, for mycobacteria, 50 µg ml^−1^ for *E. coli* (Sigma)].

### Construction of bioluminescent reporter plasmids and strains

The plasmids used in this study are described in Tables 1–4. The integrating expression vectors pMV306hsp and pMV306myc were constructed by cloning into pMV306 the promoters P*_hsp60_* and P*_myc1_tetO* obtained as NotI-HindIII and XbaI-SalI fragments from pSMT3 and pSE100 respectively. All reporter genes, except for the *lux* operon, were PCR amplified using the primers and templates listed in Table 5. These primers contained an optimised Shine-Dalgarno sequence (Mega SD) [45] and/or restriction sites as indicated. The sequence of the PCR products was confirmed by DNA sequencing. The *M. smegmatis* optimised *gluc* gene was cloned into pSMT3 and pSMT3M as a BamHI-HindIII fragment and into pMV306myc as an EcoRI-SalI fragment. For cloning the wild-type and optimised *gluc* genes into pMV306hsp the PCR products were digested with EcoRI-SalI. Plasmids containing the *M. tuberculosis* optimised *ffluc* gene were made in a similar way but cloning the EcoRI-XbaI PCR product into pUC18 first to use the restriction sites of this vector's MCS. The wild-type *ffluc* was cloned into pMV306hsp as a HindIII-SalI insert. Plasmids pMV306hsp+Lux, pSMT3+Lux and pSMT3M+Lux were created by cloning a 5.7-kb EcoRI-PstI blunted fragment (containing the whole *lux* operon) from pSB2025 into the respective expression vector. Deletion of a 0.4 kb NotI-EcoRI fragment (containing P*_hsp60_*) from pMV306hsp+Lux and insertion of P*_myc1_tetO* from pMV306myc produced pMV306myc+Lux. In a similar way, the reporter plasmids containing the G13 promoter were made replacing the P*_hsp60_* of the corresponding reporter vector with P*_G13_* by digestion with NotI-EcoRI. pMVhsp+LuxAB+G13+CDE was obtained by cloning the KpnI P*_G13_* PCR fragment into pMVhsp+Lux in front of *luxC*. Finally, *luxCDABE* from pMU1* was cloned into pMV306hsp as a 5.7-kb EcoRI PCR fragment.

**Table 1:** Plasmids used in this study.

Plasmid	Description	Reference or source
pSMT3	Mycobacterial replicating expression vector containing P*_hsp60_*, Hyg^r^	[75]
pSMT3M	pSMT3 with a high copy number mutation	[39]
pSE100	Mycobacterial replicating expression vector containing P*_myc1_tetO*, Hyg^r^	[76]
pMV306	Mycobacterial integrating vector, Km^r^	[38]
pMV306hsp	pMV306 derivative containing P*_hsp60_*	This study
pMV306myc	pMV306 derivative containing P*_myc1_tetO*	This study
pUC18	*E. coli* cloning vector, Amp^r^	Fermentas

**Table 2 pone-0010777-t002:** Firefly luciferase encoding vectors used in this study.

Plasmid	Description	Reference or source
pJ246:17659	*E. coli* cloning vector encoding the firefly luciferase (*FFluc*) codon optimized for *M. tuberculosis*, Amp^r^, Km^r^, Cm^r^, Gen^r^	J. Cirillo and K. Francis
pSMT3+FFluc	pSMT3 encoding the codon optimized *FFluc*	This study
pSMT3M+FFluc	pSMT3M encoding the codon optimized *FFluc*	This study
pMV306hsp+FFluc	pMV306hsp encoding the codon optimized *FFluc*	This study
pMV306myc+FFluc	pMV306myc encoding the codon optimized *FFluc*	This study
pMV306G13+FFluc	pMV306hsp+FFluc derivative in which P*_hsp60_* has been replaced with P*_G13_*	This study
pGL2-Basic	*E. coli* vector encoding the wild-type *FFluc*, Amp^r^	Promega
pMV306hsp+FFlucWT	pMV306hsp encoding the wild-type *FFluc*	This study

**Table 3 pone-0010777-t003:** *Gaussia* luciferase encoding vectors used in this study.

Plasmid	Description	Reference or source
pUC57+Gluc	*E. coli* vector encoding the *Gaussia* luciferase gene optimized for *M. smegmatis* codon and lacking the first 48 nt (corresponding to the secretion signal), Amp^r^	D. Agranoff
pSMT3+Gluc	pSMT3 encoding the codon optimized *Gluc*	This study
pSMT3M+Gluc	pSMT3M encoding the codon optimized *Gluc*	This study
pMV306hsp+Gluc	pMV306hsp encoding the codon optimized *Gluc*	This study
pMV306myc+Gluc	pMV306myc encoding the codon optimized *Gluc*	This study
pMV306G13+Gluc	pMV306hsp+Gluc derivative in which P*_hsp60_* has been replaced with P*_G13_*	This study
pJ201:26462	*E. coli* cloning vector encoding the *Gaussia* luciferase gene including the secretion signal (*GlucSS*) codon optimized for *M. tuberculosis*, Km^r^	DNA 2.0
pMV306hsp+GlucSS	pMV306hsp encoding *GlucSS*	This study
pUC18+GlucWT-SS	*E. coli* cloning vector encoding the wild-type *Gluc* without the secretion signal, Amp^r^	B. A. Tannous
pUC18+GlucWT+SS	*E. coli* cloning vector encoding the wild-type *Gluc* with the secretion signal, Amp^r^	B. A. Tannous
pMV306hsp+GlucWT-SS	pMV306hsp encoding the wild-type *Gluc* without the secretion signal	This study
pMV306hsp+GlucWT+SS	pMV306hsp encoding the wild-type *Gluc* with the secretion signal	This study

**Table 4:** Bacterial luciferase encoding vectors used in this study.

Plasmid	Description	Reference or source
pSB2025	*E. coli* vector containing the *LuxABCDE* operon from *P. luminescens* modified for expression in Gram positive bacteria, Amp^r^	[41]
pSMT3+Lux	pSMT3 encoding LuxABCDE	This study
pSMT3M+Lux	pSMT3M encoding LuxABCDE	This study
pMV306hsp+Lux	pMV306hsp encoding LuxABCDE	This study
pMV306myc+Lux	pMV306myc encoding LuxABCDE	This study
pMV306G13+Lux	pMV306hsp+LuxABCDE derivative in which P*_hsp60_* has been replaced with P*_G13_*	This study
pMV306hsp+LuxAB+G13+CDE	pMV306hsp+LuxABCDE derivative with P*_G13_* cloned in front of *luxC*	This study
pMU1*	Improved pMU1 (which has a promoterless *luxCDABE* optimized for high-GC [46]) with a corrected mutation in *luxD*	A. Craney & J. Nodwell

**Table 5:** Primers used in this study.

Target	Primer^a^	Template
*FFluc*	5′-*CCGAGGAATTCGGATCC* ***AGAAGGAGAAGTACCG***ATG GAAGATGCGAAGAAC-3′	pJ246:17659
	5′-*AGGCTTCTAGAG*TCACTTGCCTCCTTTCTTCGC-3′	
*FFlucWT*	5′-*AAGCTT* ***AGAAGGAGAAGTACCG***ATGGAAGACGCCAA AAAC-3′	pGL2-Basic
	5′-*GTCGAC*TTACTTTCCGCCCTTCTTGGC-3′	
*Gluc*	5′-*CCGAGGAATTCGGATCC* ***AGAAGGAGAAGTACCG***ATG AAGCCGACCGAGAAC-3′	pUC57+Gluc
	5′-*TTATATAAGCTTGTCGAC*TCAGTCGCCGCCGGCGCC-3′	
*GlucWT-SS*	5′-*CCGAGGAATTCGGATCC* ***AGAAGGAGAAGTACCG***ATG AAACCAACTGAAAAC-3′	pUC18+GlucWT-SS
	5′-*GTCGACAAGCTT*TTAATCACCACCGGCACCCTTTAT-3′	
*GlucWT+SS*	5′-*CCGAGGAATTCGGATCC* ***AGAAGGAGAAGTACCG***AT GGGAGTGAAAGTTCTTTTTG-3′	pUC18+GlucWT+SS
	5′-*GTCGACAAGCTT*TTAATCACCACCGGCACCCTTTAT-3′	
P*_G13_*	5′-*GCGGCCGC*GATCGCCACTAGCGCCGCGGT-3′	*M. marinum*
	5′-*GAATTC*TCGGTTACCAAGCGTGCATTT-3′	
	5′-*GGTACC*GATCGCCACTAGCGCCGCGGT-3′	
	5′-*GGTACC*TCGGTTACCAAGCGTGCATTT-3′	
*LuxStm*	5′-*CCGAGAATTC*CAGATCTGACTGAGTGACCAAAG-3′	pMU1*
	5′-*TGACGAATTC*TCAGCTGTTGAACGCCTGGTT-3′	

a In italics, sequence added to include restriction sites (underlined) for cloning procedures, and optimized Shine-Dalgarno sequence (in bold) [45].

Reporter strains were obtained by electroporation of reporter plasmids into *M. smegmatis* mc^2^155 or *M. tuberculosis* H37Rv as previously described [73]. Each strain was named according to the plasmid it contained. The strains transformed with an integrating vector were checked by PCR with primers amplifying the corresponding promoter and reporter gene; whereas recombinant strains with replicating vectors were confirmed by recovering the plasmid after transformation into *E. coli*.

### Bioluminescence assays

#### Luciferases substrates

Coelenterazine (Gold BioTechnology®, Inc., St. Louis, Mo., USA), the substrate for Gluc, was reconstituted in acid methanol to a concentration of 10 mM (4.238 mg ml^−1^). The substrate for FFluc, D-luciferin (Gold BioTechnology®), was prepared in distilled water at 94 mM (30 mg ml^−1^). All stocks were stored at −20°C and diluted in broth media or D-PBS (without calcium or magnesium) immediately before use. Working solutions were kept on ice in the dark during preparation.

#### Screening of transformants

After electroporation, 10 randomly selected transformants were grown in broth media. Luminescence was then measured at room temperature on a tube luminometer (Modulus™ Single Tube Multimode Reader, Turner Biosystems) by adding coelenterazine to a final concentration of 10 µM or luciferin to 470 µM, and integrating the signal over 1 s. The results are expressed as relative light units (RLU). The luminescence of Lux transformants was measured in a similar way but without adding any substrate. *M. smegmatis* Lux electroporation plates were imaged in the IVIS® Spectrum imaging system (Caliper Life Sciences, Alameda, CA) using the Living Image® software (Caliper Life Sciences) and acquiring the signal for 1 s to 30 s. *M. smegmatis* or *M. tuberculosis* transformed with the corresponding empty vector were used to measure the background luminescence.

#### Luciferase activity assays

For the integration time, substrate and kinetics assays, two or three independent cultures of each strain were grown to an optical density (OD) at 600 nm of 0.6 (mid-log phase) and each culture was measured in duplicate at 37°C with a microplate reader (Mithras LB 940, Berthold Technologies, Bad Wildbad, Germany) using MicroWin 2000 software (Berthold Technologies). When required 50 µl of substrate was injected into 50 µl of sample in a polystyrene 96-well plate and luminescence was measured for 0.1 s to 10 s after a delay of 0.1 s. For the kinetics assay luminescence was measured immediately after adding the substrate and then every 10 s or 30 s (Gluc and FFluc respectively) for 30 min. In the case of the bacterial luciferase the light output was also measured every 30 s for 30 min but without adding exogenous substrate. Cultures of *M. smegmatis* pMV306hsp were processed in parallel to each experiment and the measurements were treated as the luminescence's background. The results are expressed as RLUs.

### Study of *Gaussia* luciferase secretion

Three independent cultures of *M. smegmatis* expressing *gluc* were grown in LB-Tween to mid-log phase (OD = 0.6). A sample of 0.5 ml of each culture was centrifuged, the supernatant filtered using a 0.22 µm Ultrafree-MC centrifugal filter unit (Millipore) and the cell pellet resuspended in 0.5 ml of fresh medium. The luminescence was then measured in duplicate in samples taken from the culture, supernatant and cells using the Mithras LB 940 microplate reader as above.

### *In vivo* studies

Experiments were performed in accordance with the Animals Scientific Procedures Act (1986) and were approved by the local Ethical Review Committee. Barrier-bred female 8–12 week old Balb/c mice (Charles River UK Ltd) were anaesthetised by intraperitoneal injection of 100 mg kg^−1^ body weight ketamine (Ketaset; Fort Dodge Animal Health, Southampton, UK) and 10 mg kg^−1^ body weight xylazine (Rompun; Bayer, Newbury, Berkshire, UK) and inoculated with *M. smegmatis* or *M. tuberculosis* by endotracheal aerosol application of a total volume of 25 µl using a Microsprayer® (PennCentury, Philadelphia, PA, USA) as previously described [74].

Assessment of bioluminescence (photons s^−1^cm^−2^ steridian [sr]^−1^) from living animals was performed using an IVIS® Spectrum system (Caliper Life Sciences, Alameda, USA) which consists of a cooled charge-coupled device camera mounted on a light-tight specimen chamber. Prior to bioluminescent imaging, mice were anaesthetised with 4% isoflurane. Luciferin dissolved in sterile D-PBS was then administered to animals inoculated with FFluc expressing strains [20 µl of 15 mg ml^−1^ (47 µM) or 30 mg ml^−1^ (94 µM) luciferin via the intranasal route, or 300 mg kg^−1^ or 500 mg kg^−1^ body weight by intraperitoneal injection]. To image mice infected with Gluc expressing *M. smegmatis* 50 µl of 0.48 mM or 0.96 mM coelenterazine (prepared by diluting the 10 mM stock in sterile D-PBS just before use) was intranasally administered (10 or 20 µg per mouse respectively), or 150 µl of 0.16 mM coelenterazine via the intraperitoneal route (10 µg). Mice were placed into the camera chamber of the IVIS® Spectrum imaging system where a controlled flow of 2.5% isoflurane in air was administered through a nose cone via the IXG8 gas anaesthesia system (Caliper Life Sciences). A grayscale reference image was taken under low illumination prior to quantification of emitted photons over 30 s to 5 min, depending on signal intensity, using the software program Living Image (Caliper Life Sciences) as an overlay on Igor (Wavemetrics, Seattle, WA). For anatomical localisation, a pseudocolour image representing light intensity (blue, least intense to red, most intense) was generated using the Living Image software and superimposed over the grayscale reference image. Bioluminescence within specific regions of individual mice was also quantified using the region of interest (ROI) tool in the Living Image software program (given as photons s^−1^). Animals were imaged immediately after inoculation, to assess the success of the delivery, and 24 h post-infection. Animals inoculated with *ffluc*- or *gluc*- expressing *M. smegmatis* were imaged at different time points after substrate administration for up to 3 h.

### Statistical analysis

Statistical analyses were performed using GraphPad Prism 5.02 (GraphPad Software, San Diego, USA). Normality of data was tested by use of the D'Agostino & Pearson omnibus normality test. According to this, differences in reporter constructs activity were assessed by use of the non-parametric Kruskal-Wallis test for comparisons of groups of three. If the Kruskal–Wallis test was statistically significant, then a Dunn's multiple comparison test was employed. For groups in pairs the non-parametric Mann-Whitney test, or the t test for normal data, were used.
